# Proximity of posterior teeth to mandibular canal with 3D measurements in a population of western China

**DOI:** 10.1371/journal.pone.0344469

**Published:** 2026-04-24

**Authors:** Lu Feng, Xi Zhao, Bo Zheng, Dingming Huang, Lan Zhang

**Affiliations:** 1 Department of stomatology, People’s Hospital of Deyang City, Deyang, Sichuan, China; 2 Department of Cariology and Endodontics, State Key Laboratory of Oral Diseases, National Clinical Center for Oral Diseases, West China Hospital of Stomatology, Sichuan University, Chengdu, China; University of Puthisastra, CAMBODIA

## Abstract

**Objectives:**

The mandibular canal (MC) is an important anatomical structure that sometimes incurs injuries. Complications, such as paresthesia, neuropathic pain, and numbness, usually occur and are challenging for patients and dentists to resolve. The relationship of the MC with mandibular posterior teeth is of great clinical importance to avoid damage. The aim of this study was to evaluate the proximity of adjacent teeth to the MC in a Western Chinese population using 3D reconstruction and measurements.

**Methods:**

Cone-beam computed tomography (CBCT) images were collected in patients who met the inclusion and exclusion criteria. The 3D models of the MCs were reconstructed and imported to 3D viewing software. The root apices were identified precisely. The relative location of the MC and the shortest distance between the MC and root apex of each mandibular posterior tooth were measured in 3D. The age and sex of the patients, the tooth position, and whether the left or right mandible was affected were recorded. The relationships between distance and age, sex, mandible position and tooth position were analyzed using t-tests or analysis of variance (ANOVA).

**Results:**

In total, 125 cone-beam computed tomography (CBCT) scans were performed. The average shortest distances from the MC to the mandibular teeth were 5.7463 ± 2.3089 mm (second premolar), 7.1025 ± 2.6063 mm (first molar) and 4.5082 ± 2.6199 mm (second molar). An occurrence rate of ≤1 mm between the root tips and the MC was found in 9.7%, 0.7% and 1.7% of the measured mandibular second and first molars and mandibular second premolars, respectively. The distances in the females were significantly shorter than those in the males in all tooth positions (P < 0.05). The distances from the MC to all roots of the mandibular second molar, distal root of the first molar, distal lingual root of the first molar or root of the second premolar were smaller in the 18- to 28-year-old group than in the other age groups (P < 0.05). No significant differences were found concerning the location (right/left mandible) of the teeth (P > 0.05). More than half of the mandibular second molars were located on the lingual side of the MC, while the mandibular first molars and second premolars were mostly located on the buccal side of the MC.

**Conclusions:**

There is a strong relationship between the roots of the mandibular second molars and the MC in Western Chinese people. Distance is strongly related to sex and age. Therefore, clinicians should carefully consider these factors when performing preoperative examinations or surgical or endodontic procedures. The 3D reconstruction and measurements of CBCT images can be used to provide clinicians with a more accurate and comprehensive understanding of the relationships between the MC and adjacent structures.

## Introduction

The mandibular canal (MC) runs obliquely downward and forward along the mandible from the mandibular foramen to the mental foramen. It contains the inferior alveolar nerve (IAN), artery and vein, which form the inferior alveolar neurovascular bundle [1]. A branch of the trigeminal nerve, the IAN, innervates the sensory nerves of the lower lip, chin, teeth and mandible on the same side. The inferior alveolar vessels nourish the mandibular molar, premolar, mandible, gingiva and mucosa.

The components of the inferior alveolar neurovascular bundle from the superior to inferior direction are as follows: vein, artery, and nerve. The IAN lies below vessels; the artery is located lingual to the vein in most situations. When the MC incurs injury, hemorrhage, neuropathic pain, swelling and paresthesia in the corresponding area usually occur. Hemorrhage often occurs when the bundles are damaged during surgery. Transient numbness can be a result of compression due to hematoma [2]. In addition, unusual ocular complications such as diplopia, amaurosis, mydriasis, enophthalmos, and ophthalmoplegia can occur when local anesthetics are injected into the vessels of the MC during local anesthesia of the IAN [3].

In addition to local anesthesia, other dental procedures, such as root canal treatment [4], endodontic surgery [5], mandibular third molar extraction [6], orthognathic surgery [7]and implant surgery [8], can cause damage to the MC. It has been reported that implant surgeries account for approximately 43% of IAN injuries, and endodontic treatment occurs in approximately 4.2% of cases [9]. Another study revealed that the incidence of IAN damage related to endodontic procedures was near 8% [10]. In addition, overinstrumentation of the root canal often leads to the enlargement of root apices, causing the leakage of irrigants, medicaments and filling materials, which may cause indirect injury to the MC due to toxicity [11,12]. Sodium hypochlorite (NaOCl) and calcium hydroxide (Ca(OH)_2_) are widely used during root canal treatment and are strongly alkaline and neurotoxic. When they leak into the MC, they can cause severe neurotoxic complications [13]. Additionally, sealers and filling materials can also cause severe consequences when forced out of the root canals [14].

The major structures near the MC are the mandibular teeth. The proximity of the root apices of mandibular posterior teeth to the MC may pose a potential risk of injury during dental procedures. To minimize these risks, it is imperative to know the precise position of the MC and its relationship with the neighboring teeth.

The relationships between the MC and posterior teeth were estimated previously in a single layer of CBCT images with two-dimensional measurements, which cannot reveal the 3D position of the MC in the mandible. Some important anatomical information may have been missed by the 2D measurements, and clinicians may make mistakes when referring to such 2D measurements. In addition, some studies [15,16] have indicated that age, sex and race may affect the relationships between the MC and neighboring teeth. Thus, the aim of this study was to explore the MC location in a 3D reconstructed model with a 3D measurement method and analyze the age-related, sex-related and race-related changes in the MC locations in a Western Chinese population.

## Materials and methods

The study was approved by the ethics committee of the West China Hospital of Stomatology. All enrolled patients were informed and signed consent prior to the study. All CBCT files were saved in the digital imaging and communications in medicine (DICOM) format, which contained data of the whole mandible and teeth and were obtained from patients who visited the West China Hospital of Stomatology in recent years. The inclusion criteria were that the patient was older than 18 years and there were no missing teeth in the mandible posterior teeth region. The exclusion criteria were as follows: 1) the images were unclear, 2) the patient data had motion or metal-related artifacts, 3) the patient had a history of trauma, surgery, tumors, malformations, periapical inflammation or other lesions in the mandible, and 4) the patient had a bone metabolism or immune disease. According to the criteria, a total of 125 CBCT DICOM images from 79 patients (34 males, 45 females) were included.

The images were acquired by a 3D Accuitomo CBCT machine (MCT-1[EX-2F], J. Morita Manufacturing Corp, Kyoto, Japan), which provided a grayscale image of 14 bits and had a voxel size of 0.125 mm. All CBCT images were taken at 5.0 mA and 80 kV with an exposure time of 17 s.

(The metadata were accessed from the department of Radiological, West China Hospital of Stomatology, Sichuan University between 23/07/2018 and 31/12/2020，under ethics approval number WCHSIRB-D-2017–152.)

### 3D reconstruction and measurements

**Fig 1:**
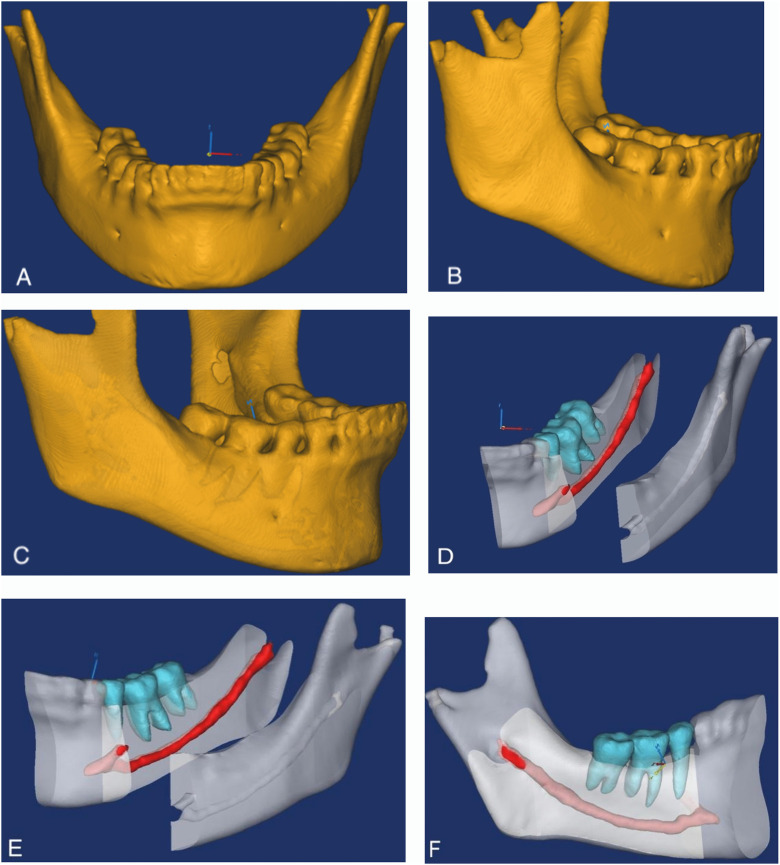
Three-dimensional model of mandible, MC and posterior teeth. A: frontal view of the mandible; B: buccal view of the mandible; C: the transparency was low, showing the relationship between the root and the mental foramen; D,E:buccal view,F:lingual view; D,E,F:showing the mandibular posterior teeth，MC and mandible with moderate transparency. The blue, red, gray area represented mandibular posterior teeth, mandibular canal and mandible respectively.

Reconstruction of the mandible, posterior teeth and MC was conducted in Mimics 17.0 software (Materialise, Leuven, Belgium) with the combination of a semiautomatic threshold-based segmentation approach, manual editing and three-dimensional arithmetic models (Fig 1). The reconstructed anatomical structures of the MC in the 3D models were made transparent by adjusting the transparency.

**Fig 2:**
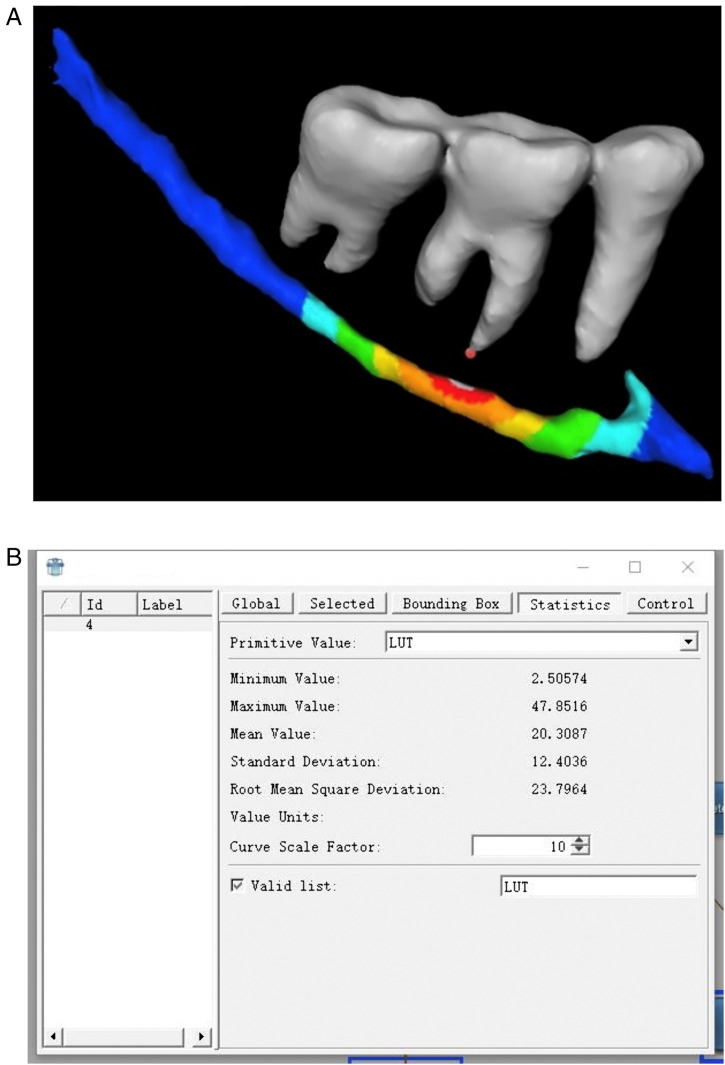
Three-dimensional measurement model of distance between posterior teeth and MC. A: the gray area is the mandibular posterior teeth, blue-green area is the MC, and red area on the MC is the region which the minimum distance appears. The red point represents the apex of mandibular first molar. B: the maximum, minimum, average and standard deviation of the distance between mandibular posterior root apices and MC.

Each root apex of the posterior teeth was precisely marked and defined according to the tooth position. The relative locations of the MC and posterior teeth were recorded according to the three-dimensional coordinate system in the software. Then, the 3D models of the MC and posterior teeth whose apices had already been tagged were imported into MeVisLab 3.1.1 software (MeVis Medical Solutions AG, Germany) (Fig 2). Then, according to the method published by Gao Yuan et al [17], the program framework was built, and the shortest distances from the MC to each of the root apices of the posterior teeth were measured. In brief, the nodes of the MC wall were 10000–15000, and the shortest distances between the MC and root apices were automatically analyzed with the measurement tool in the software (Fig 2).

Distances and related measures, such as the positions of the tooth and mesial and distal roots, were recorded in detail. The distances between the root tips and the MC were further divided into four groups. Group I included distances ≤1 mm (contacted); group II included distances 1–3 mm; group III included distances 3–5 mm; and group IV included distances >5 mm. The region that had the shortest distance from the MC was marked at the same time. In addition to these data, corresponding basic information, including age, sex and the location of the mandible, was also acquired.

### Statistical analysis

The shortest distance from each root apex to the MC was recorded. In addition, the relationships of shortest distance with the mandible position, patient age and patient sex were analyzed. Statistically significant differences were evaluated with t-test or analysis of variance (ANOVA) in SPSS 23.0. P values less than 0.05 were considered statistically significant.

## Results

**Fig 3:**
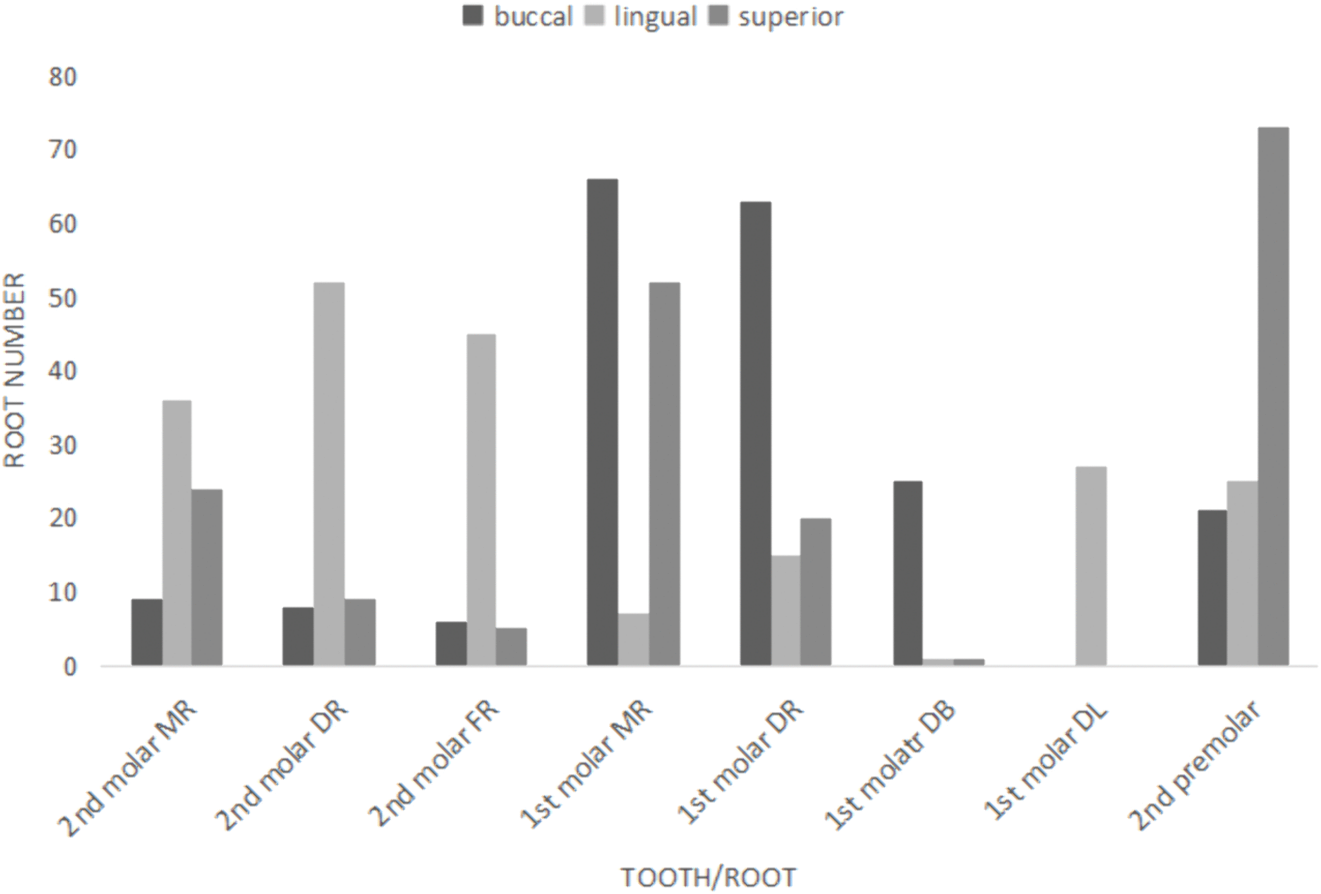
Relative location of MC and root of mandibular posterior teeth. MR, mesial root; DR, distal root; DB, distal buccal root; DL, distal lingual root; FR: fused root.

Thirty-four males and 45 females were involved in the study, which included 119 mandibular first premolars, 277 mandibular first molars and 194 mandibular second molars. Most roots of the mandibular second molars were located on the lingual side of the MC, while the roots of the first molars were on the buccal side of the MC (Fig 3).

The average shortest distance from the mandibular tooth apices to the MC (Table 1) was 5.7463 ± 2.3089 mm for the second premolar, 7.1025 ± 2.6063 mm for the first molar and 4.5082 ± 2.6199 mm for the second molar. For the second molar, the distance to the MC was 5.1107 ± 2.5183 mm from the mesial root, 4.6502 ± 2.6992 mm from the distal root, and 3.4464 ± 2.2305 mm from the fused second molar. For the first molar, the distance was 7.0472 ± 2.4423 mm from the mesial root, 6.6167 ± 2.5361 mm from the distal root, 7.1286 ± 2.6690 mm from the distal buccal root and 9.0962 ± 2.7881 mm from the distal lingual root.

**Table 1:** Detailed data of the distance from the root apices to the MC.

Tooth/root	n	min	max	mean	sd
2nd molar	194	−0.3489	12.7730	4.5082	2.6199
mesial root	69	0.2693	11.0673	5.1107	2.5183
distal root	69	0.1835	12.7730	4.6502	2.6992
fused root	56	−0.3489	7.7855	3.4464	2.2305
1st molar	277	0.7548	15.8416	7.1025	2.6063
mesial root	125	0.7548	12.4946	7.0472	2.4423
distal root	98	0.9861	11.6656	6.6167	2.5361
distal buccal root	27	2.0025	12.8363	7.1286	2.6690
Distal lingual root	27	5.2890	15.8416	9.0962	2.7881
2nd premolar	119	0.2634	11.8597	5.7463	2.3089
total	590	−0.3489	15.8416	6.0355	2.7884

Group I distances (≤1 mm) between the root tips and the MC were found in 9.7%, 0.7% and 1.7% of the mandibular second and first molars and mandibular second premolars, respectively.

**Fig 4:**
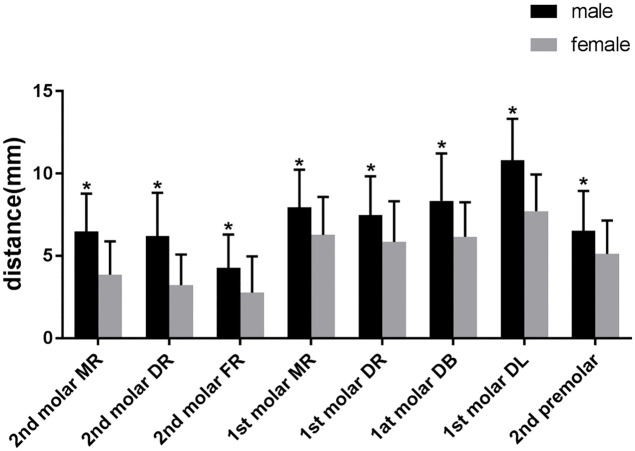
The relationship of gender and distance between the root apices and MC. MR, mesial root; DR, distal root; FR, fused root; DB, distal buccal root; DL, distal lingual root.*p < 0.05.Error bars = standard error of the mean(SEM).

The distances from the root apexes to the MC were significantly shorter in the females than in the males regardless of the tooth position (P < 0.05) (Fig 4).

**Fig 5:**
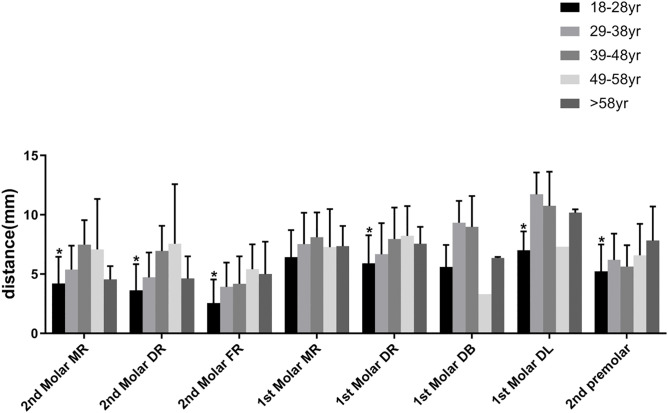
The relationship of age and distance between root apices and MC. MR, mesial root; DR, distal root; FR, fused root; DB, distal buccal root; DL, distal lingual root. *p < 0.05.Error bars = standard error of the mean (SEM).

To determined the influence of patient age on the relationships of the distances between the root apices and the MC, the patients aged 18–66 years were additionally divided into the following groups (Fig 5): 18–28 years, 29–38 years, 39–48 years, 49–58 years, and >58 years. The mean distances from the MC to the mandibular second molar, distal roots and distal lingual roots of the first molar and second premolar were significantly shorter in the 18- to 28-year-old group than in the other groups (P < 0.05) (Fig 5).

**Fig 6:**
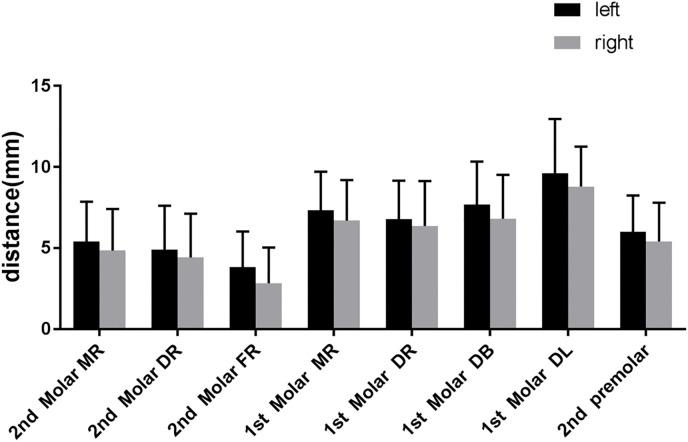
The relationship of mandible position and distance between the root apices and MC. MR, mesial root; DR, distal root; FR, fused root; DB, distal buccal root; DL, distal lingual root.*p < 0.05.Error bars = standard error of the mean(SEM).

No significant differences were found in the distances between the left and right mandibular posterior teeth and the MC (P > 0.05) (Fig 6).

## Discussion

Distances are rarely measured with dry mandibles, periapical radiographs and panoramic radiographs. Dry mandibles need to be stored in specific conditions. It is hard to gather, and information on patients is lacking [18]. Observing the mandibular canal on periapical radiographs is difficult, especially in patients with a small mouth, malpositioned teeth and large mandibular tori. These conditions may prevent proper placement of the film for exposure of the mandibular canal [19]. However, panoramic radiographs cannot provide clear visibility and have magnification and distortion issues in some situations [20].

With more accuracy and less manifestation, CBCT images have been widely used to assess the location of the MC and its relationship with mandibular posterior teeth preoperatively. However, measurements from one CBCT image cannot provide continuous information on the MC or the relationships with adjacent anatomical structures, and important anatomical information may be missed before treatment, causing severe damage.

3D reconstruction of the MC, neighboring mandible and teeth using CBCT images can provide clinicians visual and consecutive information about the location of the MC and its proximity with neighboring teeth, forming a trajectory in the body of mandible. The present study used 3D models to obtain more detailed information on the characteristics of the MC and measure the distance between the MC and mandibular posterior teeth.

In this study, the second premolar and first molar were located on the buccal side of the MC, while the root of the second molar was located on the lingual side when the MC moved posteriorly, which was consistent with the results of a study conducted by Denio et al [21] with a mature mandible. Hence, in intentional replantation surgery of mandibular second molars, the long axis of the tooth can move toward the lingual side during the extraction and insertion of the tooth to avoid injury to the MC due to the relative location. In addition, performing extraction in a controlled, prolonged manner with a slow rocking motion can prevent and mitigate potential damage to the MC and periodontal ligament cells [22].

The results indicated that in a Western Chinese population, the mandibular second molar was the closest to the MC among the measured teeth, with an average distance of 4.50 ± 2.61 mm and distances ranging from −0.34 mm to 12.77 mm. In the mandibular second molar, the distal root and fused second molar were closer to the MC. Denio et al [21] found that the mandibular second molar was the closest to the MC in 22 mandibles that were sectioned. Tyler Kovisto et al [23] also measured the distance between the root apices of the mandibular posterior teeth and the MC using CBCT and evaluated the effects of age and sex. In all groups, the root apices of the mandibular second molars were closer than were the root apices from the mandibular first molars or second premolars. In addition, the distal roots of the mandibular second molars had shorter distances than did the other roots of the first molars and second premolars. Bürklein et al [16] researched the relationship between the root apices of the mandibular posterior teeth in a German population and found that the second mandibular molar had the shortest distance, with an average distance of 3.1 mm. Chong B et al [24] revealed that apart from the third molar, the second molar was closest to MC, with a distance less than 3 mm. Although the values were different, the trend that the mandibular second molars and distal roots of the second molars were nearer to the MC than were the other roots was consistent with our results. The inconsistency in values may be due to differences in race, the observation method and/or the number of patients.

Apart from the tooth position, sex and age can also affect the distance in Western Chinese people. Our results indicated that females had shorter distances, which may explain why endodontic-related IAN injuries often occur in female patients [9]. Previous studies [15,16,25] have assessed the differences in males and females separately in German, American and British populations. The authors found that regardless of the patient’s age and tooth position, the distances in the females were always shorter than those in the males. Our results are consistent with these results. This sex difference may be due to males having a larger mandible; therefore, the mandibular teeth are farther from the MC in males than in females.

Another finding of this study was that the 18- to 28-year-old group had a smaller distance between the MC and the mandibular posterior teeth. As age increased, the distance increased, and tooth eruption and reconstruction of the mandible may have moved the MC farther away from the posterior teeth, which is consistent with findings of the studies of Bürklein et al [16] and Tyler Kovisto et al [23]. Bürklein et al [16]revealed that the distance was shorter in patients under 35 years old. Tyler Kovisto [23] indicated that patients younger than 18 had a smaller distance than did those in other age groups. Simonton et al [15,26] found that the distance between the MC and posterior teeth of the mandible increased between the ages of approximately 40–50 years and then decreased as age increased.

It is important to know that the distances in females and patients aged 18–28 years are shorter, as this information may help us make favorable decisions before treatment and during clinical operations. This location information is of great importance in endodontic treatment.

When performing tooth extraction in immediate implant surgery, the proximity between the tooth and MC is a crucial factor influencing the incidence of IAN and vessel injury. Some cases [27] of implants or drills intruding into the MC during immediate implant surgery have already been reported. In these situations, the anatomical structure of the MC itself and its relationship with adjacent structures play an important role. A good way to prevent this damage is to have clear three-dimensional vision of MC and neighboring structures. This can be achieved by 3D reconstruction and measurements. In addition, information assessed by visual and accurate 3D reconstruction and measurements may have a positive influence in avoiding or reducing injury.

3D reconstruction of CBCT images and 3D measurements overcome the limitations of CBCT 2D images and do not require invasive procedures to assess the relationship of the MC with adjacent teeth in vivo. The 3D features and measurements reported in the study can be used by clinicians to assess the MC and its relationships with adjacent teeth more precisely and directly when conducting preoperative examinations.
